# Ethnology and Archeology at the Fair

**Published:** 1900-08

**Authors:** A. L. Benedict

**Affiliations:** Supt. of Ethnology and Archeology. Buffalo, N. Y.


### Correspondence.

### ETHNOLOGY AND ARCHEOLOGY AT THE FAIR.

*To the Editor of the Buffalo Medical Journal:*

SIR.—The Pan-American Exposition has seen fit to entrust the care of the department of ethnology and archeology to a practising physician. I should be very glad if you would allow me to reach your readers with the following request for assistance.

Many members of the medical profession are interested in the study of American ethnology and archeology and not a few have valuable collections of Indian relics and skeletons from Indian graves. Those not directly interested in this study are so circumstanced as to be aware of the hobbies of their neighbors and could doubtless furnish the address of collectors. I should be greatly obliged for information and for the loan of collections for the use of this department of the Exposition. Exhibits which represent study in some special line of American ethnology and archeology will be particularly suitable.

Very truly yours,

A. L. BENEDICT, M.D.,
*Supt. of Ethnology and Archeology.*

BUFFALO, N. Y., July 3, 1900.

